# Annular rupture during transcatheter aortic valve replacement in a long-term corticosteroid user: a case report

**DOI:** 10.1093/jscr/rjad317

**Published:** 2023-05-30

**Authors:** Hironobu Nishiori, Kaoru Matsuura, Yasunori Yakita, Tomoyoshi Kanda, Hideki Kitahara, Daichi Yamashita, Yoshio Kobayashi, Goro Matsumiya

**Affiliations:** Department of Cardiovascular Surgery, Chiba University Hospital, Chiba, Japan; Department of Cardiovascular Surgery, Chiba University Hospital, Chiba, Japan; Department of Cardiovascular Surgery, Chiba University Hospital, Chiba, Japan; Department of Cardiovascular Surgery, Chiba University Hospital, Chiba, Japan; Department of Cardiology, Chiba University Hospital, Chiba, Japan; Department of Cardiology, Chiba University Hospital, Chiba, Japan; Department of Cardiology, Chiba University Hospital, Chiba, Japan; Department of Cardiovascular Surgery, Chiba University Hospital, Chiba, Japan

## Abstract

A 74-year-old woman with a history of interstitial pneumonia, who had been taking oral corticosteroids for the past 9 years, was diagnosed with severe aortic stenosis. The patient underwent transfemoral transcatheter aortic valve replacement (TAVR) with a balloon-expandable valve under local anesthesia. After deploying a 26-mm SAPIEN 3 valve with 1.5 ml less balloon inflation, transesophageal echocardiography revealed a hemorrhage in the aortic annulus. Intraoperative angiography revealed a small contrast leakage around the ascending aorta. Emergent surgical aortic valve replacement was performed successfully, with a tear at the non-left commissure closed using interrupted sutures. The patient was discharged on postoperative day 14 with no paravalvular leakage. Chronic corticosteroid use may be a risk factor for annular ruptures during TAVR. Careful balloon dilation may be necessary, especially when balloon-expandable valves are used in patients receiving long-term corticosteroids.

## INTRODUCTION

Annular rupture is a fatal complication of transcatheter aortic valve replacement (TAVR), which occurs in 0.4–2.3% of cases and requires immediate intervention [1]. Although long-term corticosteroid use is known to cause tissue fragility, the association between corticosteroid use and annular rupture during TAVR is not fully understood. Herein, we report a case of successful surgical repair of an annular rupture that occurred despite adequate precautions during TAVR in a long-term steroid user.

## CASE REPORT

**Figure 1:**
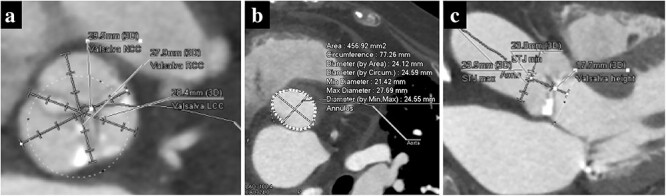
Preoperative computed tomography imaging showing a moderately calcified aortic annulus (**a**), an annulus area of 457 mm^2^ (**b**) and the short height of Valsalva (**c**).

A 74-year-old woman with a history of interstitial pneumonia was admitted to the hospital with the chief complaint of dyspnea. The patient had been taking oral prednisolone (13 mg) for 9 years and home oxygen therapy for 6 years for interstitial pneumonia. Transthoracic echocardiography (TTE) showed an aortic valve area of 0.58 cm^2^, transvalvular peak velocity of 3.6 m/s, mean transvalvular pressure gradient of 28 mmHg and left ventricular ejection fraction of 33%. The patient was diagnosed with severe aortic stenosis. Computed tomography revealed a tricuspid aortic valve with an area of 456.9 mm^2^ and basal ring circumference of 77.3 mm, with a mildly calcified aortic annulus (Fig. 1a and b). We decided to perform transfemoral TAVR under local anesthesia. We chose the 26-mm SAPIEN 3 valve (Edwards Lifesciences, Irvine, CA, USA) for the following reasons: mild aortic annulus calcification, the possibility of future TAVR explants or transcatheter aortic valve in the transcatheter aortic valve (TAV-in-TAV) considering the patient’s age, and the fact that the self-expandable valves (SEVs) were unsuitable because of the small size of the sinus of Valsalva (Fig. 1c).

**Figure 2:**
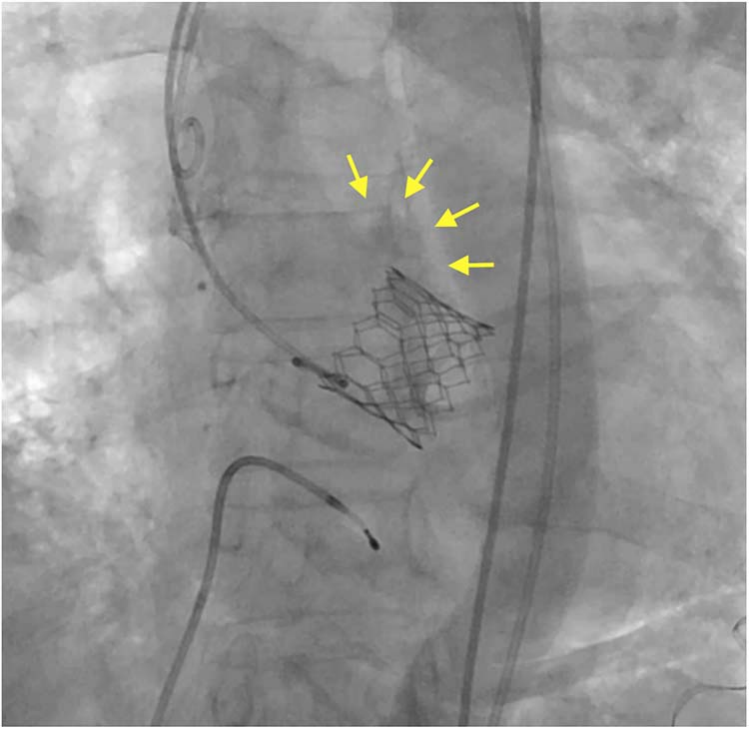
Intraoperative angiography shows a small contrast leakage at the ascending aorta (arrows).

**Figure 3:**
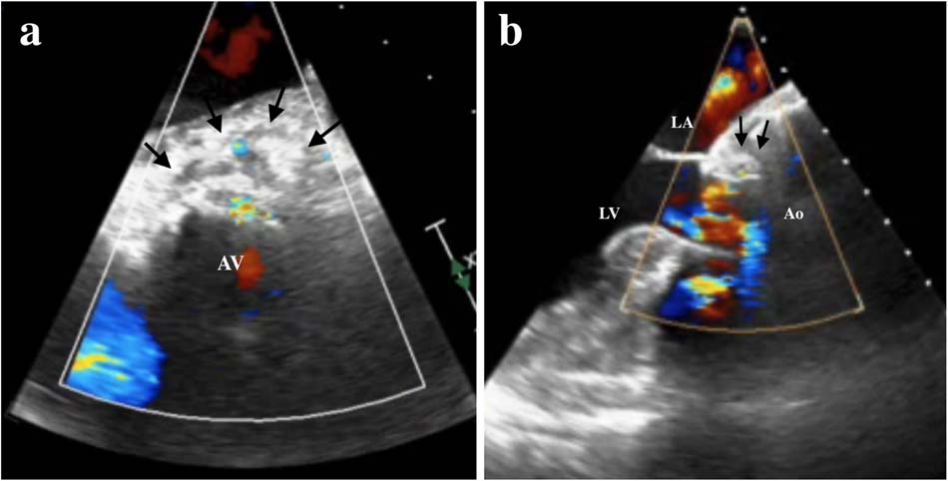
TEE showing the hematoma around the ascending aorta and blood flow at the hematoma at 0 degrees of view (**a**, arrows), at 120 degrees of view (**b**, arrows). LA, left atrium; LV, left ventricle; Ao, aorta; AV, aortic valve.

**Figure 4:**
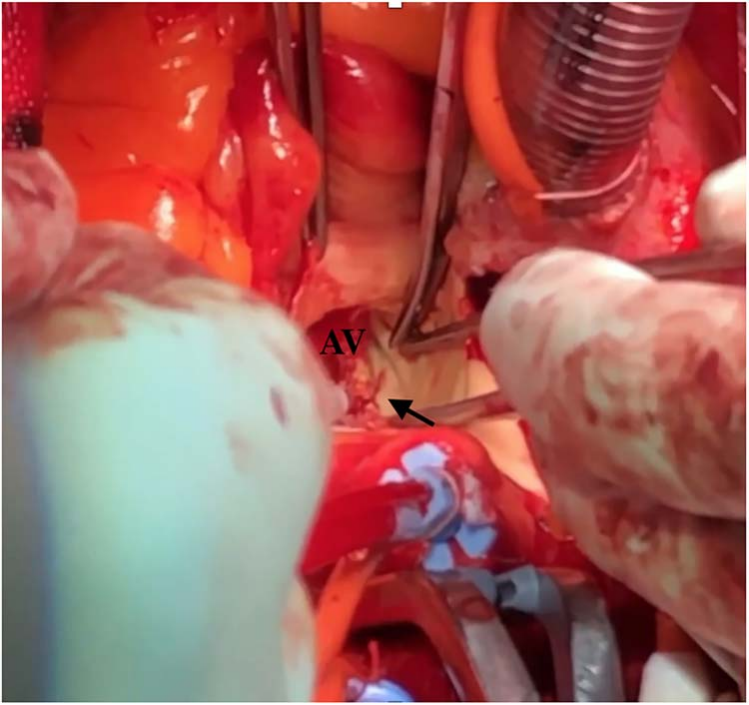
Intraoperative finding shows a tear at the left non-commissure. AV: aortic valve.

Under light sedation and local anesthesia, a 26-mm SAPIEN 3 valve was deployed with 1.5 ml less balloon inflation to avoid excessive pressure on the aortic annulus. After valve deployment, TTE revealed a small pericardial effusion. Pericardial drainage was performed, and blood was drained. Intraoperative angiography revealed a small amount of contrast leakage around the ascending aorta (Fig. 2). Transesophageal echocardiography (TEE) under deep sedation revealed a hematoma at the aortic annulus with blood inflow (Fig. 3a and b). Emergent open surgery was performed. The aorta was exposed through a median sternotomy. Cardiopulmonary bypass was established via the ascending aorta and the right atrium, and the aorta was cross-clamped. Antegrade cardioplegia was administered via an aortic root cannula, and cardiac arrest was achieved without complications. The prosthetic valve was firmly adherent to the aortic annulus and carefully removed. A tear in the non-left coronary commissure was sutured using 5–0 Prolene (Fig. 4). An INSPIRIS 21 mm valve (Edwards Lifesciences, Irvine, CA, USA) was implanted. The patient was discharged on postoperative day 14 after rehabilitation. One year after surgery, TTE revealed no paravalvular leakage and a preserved ejection fraction of 34%.

## DISCUSSION

Annular rupture is one of the most serious complications of TAVR, with a poor prognosis with a 49–67% mortality rate [1]. Several risk factors for annular rupture during TAVR are reported, including female sex, small body surface area, degree and location of subannular calcification, balloon-expandable valves (BEVs), and oversizing valves (>20%) [2]. Chronic use of systemic corticosteroids is known to increase tissue fragility, including that of the vessel wall, and coronary artery perforation rate during percutaneous coronary intervention [3]. Few reports have examined whether chronic steroid use is a causative factor of annular rupture during TAVR. In the Optimized Catheter Vascular Intervention Structural Heart Disease Registry in Japan, the incidence of annular rupture among 56 corticosteroid users and non-corticosteroid users was 3 and 1%, respectively, with no significant difference [4]. Joshi *et al.* reported a 2.0% incidence rate of annular rupture during TAVR in 99 steroid users, which was significantly higher than that in nonsteroid users [5]. The chronic corticosteroid use can cause delayed wound healing and worsening of the underlying disease during the perioperative period in open cardiac surgery [4], and the Society of Thoracic Surgeons lists steroid therapy as a risk factor for increased mortality in cardiac surgery [6]. Corticosteroid users are more likely to choose TAVR over surgical aortic valve replacement. However, we should note that the incidence of TAVR-specific complications, such as annular rupture, may increase in long-term corticosteroid users.

The BEV has several advantages over SEV: lower incidence of complete atrioventricular block, easier to perform percutaneous coronary intervention or TAV-in-TAV in the future, less frequency of concomitant aortic repair at TAVR explant and easier to perform in cases with horizontal aorta using the flexion of the distal catheter mechanism [7, 8]. In addition, SEV is not anatomically compatible with the small size of the sinus of Valsalva, as in this case. Although BEV has the significant disadvantage of an 11-fold higher incidence of annular rupture [2], the above characteristics make using BEV preferable in some situations. In this case, the BEV size was 111% of the aortic annulus diameter, which was appropriate [2]. To prevent annular rupture during TAVR using BEV, an incomplete inflation strategy with 2–3 ml less balloon inflation has been reported [9]. In this case, an even smaller balloon volume than the 1.5 ml less balloon inflation might have been acceptable to prevent annular rupture. Alternatively, another smaller 26 mm SAPIEN 3 valve could have been selected.

TEE is a great modality as an initial approach to diagnose annular rupture and to define the extent of aortic root disruption during TAVR. Because the valve seals the rupture, intra-annular rupture during TAVR may be clinically undetectable in some cases [1]. In this case, TEE was performed promptly after deep sedation, and annular rupture was detected, allowing emergent surgery. The surgical technique of intra-annular rupture during TAVR has been reported to repair a ruptured aortic annulus with an autologous pericardial patch [1]. In this case, the tear was small and noticeable and was closed using interrupted sutures.

We report a case of TAVR using BEV in a long-term corticosteroid user that resulted in annular rupture and subsequent surgical repair. Considering tissue fragility in corticosteroid users, careful balloon dilation may be necessary when using BEV.
